# A social innovation to empower community-led monitoring and mobilization for HIV prevention in rural Kenya: experimenting to reduce the HIV prevention policy-implementation gap

**DOI:** 10.3389/fpubh.2023.1240200

**Published:** 2023-11-03

**Authors:** Michael Goodman, Janet Turan, Philip Keiser, Sarah Seidel, Lauren Raimer-Goodman, Stanley Gitari, Fridah Mukiri, Marie Brault, Premal Patel

**Affiliations:** ^1^Department of Internal Medicine, The University of Texas Medical Branch, Galveston, TX, United States; ^2^Department of Global Health and Emerging Diseases, The University of Texas Medical Branch School of Public and Population Health, Galveston, TX, United States; ^3^Department of Health Policy and Organization, School of Public Health, The University of Alabama at Birmingham (UAB), Birmingham, AL, United States; ^4^Sodzo International, Houston, TX, United States; ^5^Department of Pediatrics, The University of Texas Medical Branch, Galveston, TX, United States; ^6^Sodzo Kenya, Maua, Meru Co., Kenya; ^7^Department of Health Promotion and Behavioral Sciences, The University of Texas Health Science Center School of Public Health, Houston, TX, United States

**Keywords:** HIV-related stigma, HIV testing, collective efficacy, positive emotions, Kenya

## Abstract

**Introduction:**

Strong policy guidance has recently emerged identifying focal points at multiple levels and across sectors to end the persistent HIV pandemic and related inequities. Reducing the policy-implementation gap, as with the evidence-policy gap, requires strategic alignment between interventional research and policy realms. Global- and national-level HIV policy indicate a need for community-led efforts to reduce HIV stigma, and increase uptake of HIV prevention tools.

**Methods:**

This study assesses a process-driven approach to facilitating community-led efforts to reduce HIV stigma, and build a generative context for community-led HIV prevention. The study intervention combines an adapted group-based microfinance process, a novel psychological curriculum, and leadership development at a scale now involving over 10,000 rural Kenyans across 39 villages.

**Results:**

Consistent with interventional goals, and current relevant psychosocial theories, we find collective emotion, and HIV stigma (blame and discrimination) significantly improve with more time participating in the in the program and novel curriculum. Further, HIV stigma predicts subsequent reporting of ever being tested for HIV, and the intervention led to the development of “HIV prevention resource committees” – groups of participants committed to undergo training to reduce HIV stigma and prevent HIV within their communities.

**Discussion:**

Implications for further research to reduce the HIV policy-implementation gap are discussed, directly within this interventional context and more generally.

## Introduction

To end the AIDS pandemic by the end of the decade, global actors need to commit to several important goals. The UNGA declaration outlines these goals, which include transforming inequalities that contribute to new HIV cases, promoting community engagement and leadership, emphasizing HIV prevention, empowering women, and reducing HIV stigma (1). These commitments are reflected in the 2022 Kenyan National AIDS and STI Control Programs conclusions from a recent analysis of the population (2). Unfortunately, less than 80% of people living with HIV in Kenya are aware of their status, which falls short of the lowest of the 95–95-95 goals. The Kenyan strategy aims to improve this situation by aligning health systems with community-led efforts, identifying individuals who have not been tested through community engagement, using group-based testing methods, promoting health literacy, and reducing HIV stigma. Table 1 summarizes these commitments.

**Table 1:** Global and Kenyan national HIV policy priority areas.

UNGA policy commitments, 2021	Kenya HIV strategic commitments, 2022
· Transformative action to end social, economic, racial and gender inequalities;	· Align health systems with community-led efforts;
· Enacting meaningful community engagement toward comprehensive prevention, treatment, care and support for people living with HIV;	· Engage communities to identify individuals who have not been tested;
· Re-committing to the highest attainable health for all;	· Shift to community- and group-based testing modalities and health literacy promotion; and
· Focusing on women, adolescent girls and children in the 41 countries containing 90 percent of people newly infected;	· Reduce HIV Stigma & discrimination
· Establishing prevention as the cornerstone of an effective HIV response;	
· Scaling scientifically accurate health information through public awareness campaigns and targeted HIV education;	
· Reaching the 95-95-95 goals (% of people living with HIV aware of their status; % of people living with HIV who have been diagnosed and are on treatment; % of people living with HIV on treatment with viral suppression);	
· Differentiating service-delivery models, including articulating effective community-led and -based services;	
· Gender empowerment; and	
· Reducing HIV stigma and discrimination	
Selected priorities listed by UNGA and Kenya HIV strategy.	

The policies recognize the need for innovative intervention strategies to end the HIV pandemic. These strategies should prioritize community-based initiatives and facilitate connections between community leaders and testing, prevention, and treatment services. Addressing HIV stigma at the community level is also critical, as research has shown that stigma can discourage individuals from seeking testing or discussing treatment options with healthcare providers (3–7). Despite the emergence of advanced HIV treatments, stigma remains a significant barrier to effective care and communication (8, 9). Therefore, reducing HIV stigma at the community level is crucial to ending the pandemic. The policy frameworks are valuable in highlighting the importance of HIV stigma reduction, promoting equity and community-based development, and fostering partnerships between community leaders and healthcare providers.

In order for the global HIV community to make progress toward equality, stigma elimination, and ending the HIV pandemic, we need to take proactive steps to bridge the gap between policy and implementation. Hudson et al. identify four effective models for implementing policy, with this study focusing on experimental implementation (10). This approach differs from dominant top-down approaches that only focus on HIV treatment, seeking to reduce duplicative resource allocation, encourage community-led development, reduce HIV stigma, empower disenfranchised groups, and connect with existing clinical care options (11–14). To simplify the bottom-up approach, experimental implementation efforts must show measurable and replicable mechanisms of action. Within experimental implementation, it is crucial to demonstrate how to foster community leadership, decrease HIV stigma, promote HIV testing and prevention, and work with the current HIV health system resources.

To improve HIV prevention and bridge the policy-implementation gap, we need to simplify intervention methods and mechanisms that can support community leadership in preventing HIV through reducing stigma, conducting HIV testing, engaging in prevention activities, and collaborating with existing health system resources. New approaches that integrate community-led actions with current HIV clinical resources will require breaking down disciplinary barriers (such as clinical treatment or community-engaged research) to better address the biopsychosocial aspects of the HIV pandemic. This movement will require a deeper exchange of knowledge between biomedical and social sciences within a public health framework (15, 16).

### The Flourishing Community Model

This report presents the preliminary findings of a community intervention in Kenya known as the “flourishing community” model. Its overarching objective is to foster sustainable well-being at the individual, family, and community levels (17), encompassing economic, social, psychological, behavioral, and physical dimensions. More detailed accounts of the intervention are available elsewhere (18). The project has yielded favorable outcomes, including enhanced mental health, water and food security, cooperation among intimate partners, and improved parenting practices among its participants (19–22). Evidence from a related program that seeks to reintegrate street-involved children and youth indicates that family involvement in the flourishing community model intervention is linked to interventional success (23). Currently, the project caters to more than 10,000 families weekly across 39 villages, at an annual cost of less than $10 per family. The intervention is structured to foster personal, group, and village autonomy and self-governance, with microfinance groups serving as a means of cultivating trust and mutual reliance to identify values and actions that further these objectives.

### Setting the stage

The intervention process starts by identifying families that are vulnerable due to various conditions. This identification is done either by retracing families of children living on the streets or through families identified from HIV clinics. Next, these families are invited to form savings and internal-lending groups with their neighbors. The lending activities of the group depend on defined processes with specific points for members to provide input such as the amount to be lent, terms of lending, grou*p* values, and motto. The demand for group lending activities is high enough that within a village setting, only one or a few focal families are identified before word-of-mouth yields greater collective interest. The village-level enrollment grows from one group of 30 families to over 1,000 families within 1 or 2 years. Group members take turns providing leadership of weekly activities and discussion topics, providing opportunities to improve joint behaviors and encourage shared group leadership. After groups are formalized and stable, members are invited to nominate one or two members to join a “Parliamentary Committee” that reflects concerns of group families and the larger community. This committee provides guidance to new groups, groups-in-conflict, and advocates on behalf of group concerns.

The Flourishing Community model integrates theoretical and empirical findings from an emerging body of research that can be largely organized under multilevel theories of behavioral and cultural evolution (24, 25). In short, human individuals adapt to new and changing environments through varying, selecting and retaining affective, cognitive, motivational, attentional, and overt behavioral states conducive to optimal well-being within respective contexts (26). Human communities adapt to new and changing environments through prosocial activities – like coordinating activities, collective action, and building mutual trust and identity (27). The question of “flourishing” involves the extent to which individuals and groups can intentionally select and retain various states (i.e., overt behavioral, affective, cognitive, etc.) conducive to autonomous, valued growth (28). Of particular theoretical utility to the challenge of fostering community-led action to prevent HIV, biased attitudes toward outsiders (e.g., people with the “HIV positive” label) can be reduced through promoting secure social relationships (29). Secure social relationships can be enhanced through cultivating feelings of peace, safety, gratitude and happiness (30).

The present study explores the dynamic between HIV stigma, secure social relations – in particular the belief in shared capacity to address salient challenges (collective efficacy) – and positive emotions – routine feelings of peace, safety, gratitude and happiness. The general question is whether lower HIV stigma is temporally correlated with collective efficacy, a common product of group-based microfinance programs, or positive emotions – potentially through a “broaden and build” dynamic promoting awareness and more inclusive attitudes toward people living with HIV. This dynamic would be consistent with other research showing discrimination toward other people can be reduced by the cultivation of positive emotions and more secure relationships (29).

### Pilot testing the Pathways to Flourishing Curriculum

We recently introduced and pilot tested a novel 6-month curriculum integrating evidence-based elements from positive psychology, compassion-based acceptance and commitment training, and group-based interpersonal therapy for depression. The Pathways to Flourishing (“P2F”) curriculum aims to foster psychological flexibility and adaptive coping resources, compassion for self and others, curiosity, positive affect, gratitude, meaning in life, autonomy, intrinsic motivation and self-determination generally, decreasing depression and anxiety. Far different to an effort to promote exchanging concerns for challenges with generally positive outlook, the P2F curriculum aims to strengthen social and psychological components that foster engagement to improve these challenges in on-going, “upward spirals” of personal, familial, and communal growth (31, 32). Participants who finish participating in the P2F curriculum are invited to identify specific areas where they would like to see improvements within their community, and to form “Resource Committees” with participants with similar concerns. This approach is described in a previous publication, though this process was documented before we introduced the P2F curriculum (16).

### Flourishing community tree metaphor

**Figure 1:**
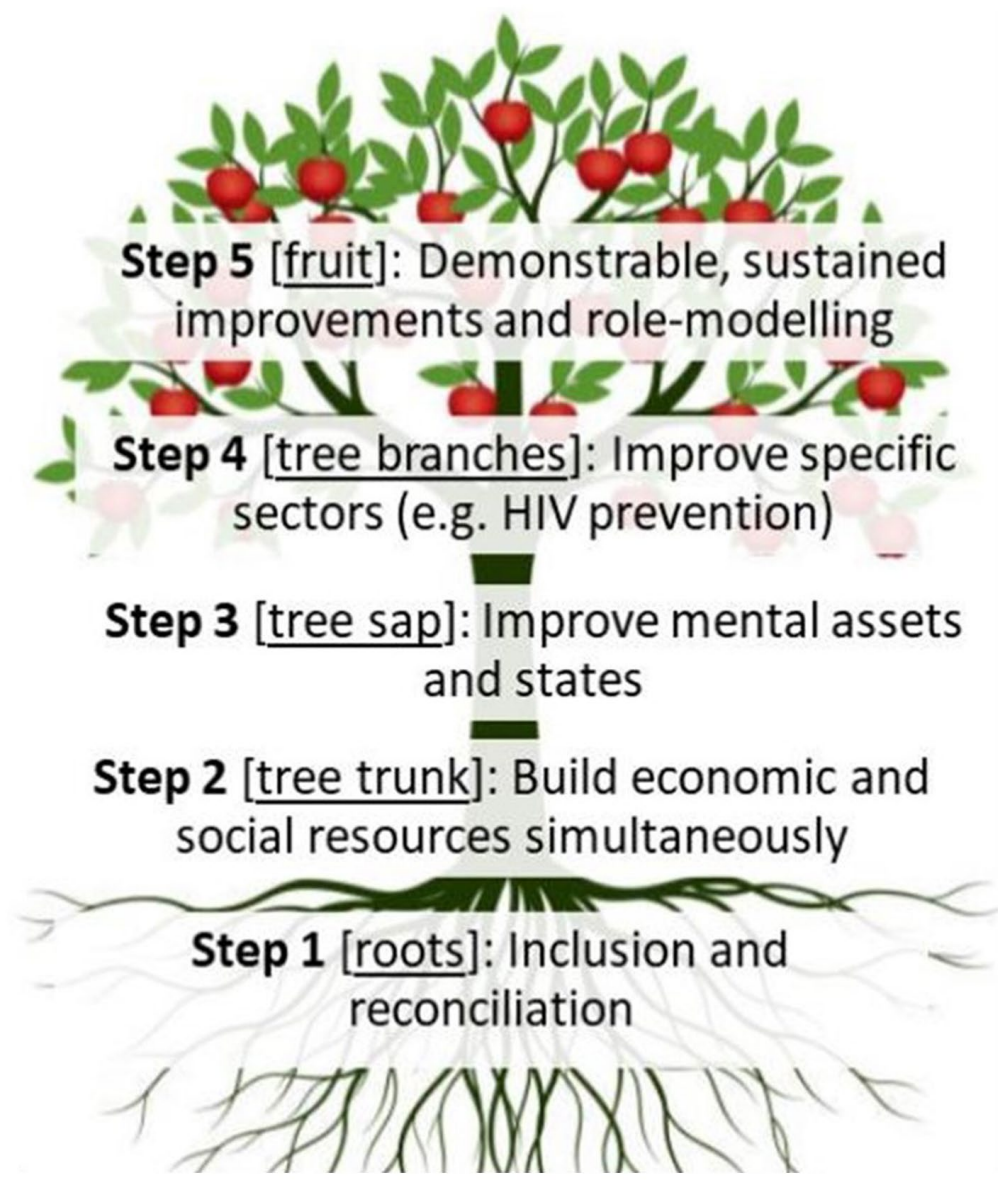
Flourishing communities tree metaphor.

Rather than requiring the lengthy and nuanced psychosocial and cultural frameworks through which the Flourishing Community model was co-developed in transformative praxis with communities (33), the metaphor of a fruiting tree has emerged to conceptualize the dynamics of the Flourishing Community model. As shown in Figure 1, the Flourishing Community model can be conceptualized as a fruit tree. The roots represent bringing together community members who previously may have been estranged (Step 1). The tree trunk represents the structure of the community – specifically, using the savings- and internal-lending model to build social connections, reciprocity, and belonging (step 2). The tree sap represents improved mental assets and states (e.g., sense of hope and purpose; reduced depression and anxiety), as improved mental conditions promote positive behavioral and attitudinal adaptation to new situations (step 3). The P2F curriculum specifically focuses on improving step 3. The branches, leaves and flowers represent growing in specific directions (e.g., HIV prevention), identifying supportive resources outside of the community (e.g., HIV testing, clinical care), and utilizing these resources to grow – as leaves photosynthesize sunlight and flowers permit pollination (step 4). Step 4 is the point at which Resource Committees emerge to focus communal concern around specific areas – like linking with HIV testing resources. The fruit from a flourishing community will be tangible, identifiable, and create the potential for lasting change – as a seed permits new life (step 5).

### Study Aim

This study includes two sets of inferential analyses. In the primary analysis, we analyze temporal relationships within and between positive emotion, collective efficacy, and HIV stigma among 2 year-long cohorts of participants in a community-based intervention to reduce the HIV prevention policy-implementation gap in Kenya. One cohort participated in the pilot test of the Pathways to Flourishing curriculum and group-based microfinance programming, while the other cohort participated in the group-based microfinance programming only. This study does not aim to compare variables between the two assessed cohorts.

In the secondary analysis, we assess whether later reports of ever testing for HIV were predicted by prior reported HIV stigma.

## Methods

### Intervention setting

The program currently operates in 39 villages in Meru County Kenya, with villages beginning the program since 2017 and ongoing. The locations (*k* = 10) selected for this study were chosen based on when the intervention was introduced within the village. Recruitment to the program within the village occurred within 4 months of baseline data collection for this study. Four villages were selected through a random number generator to receive the P2F curriculum pilot. The national HIV prevalence is 6.6% among women, and 3.1% among men – within Meru County, these figures are lower at 4.4 and 2.3%, respectively (2).

### Study participants

Study participants were initially sampled and interviewed in November 2021 during weekly program meetings. Seven participants within each group were subsequently randomly selected using labeled slips of paper within an opaque envelope, with 7 slips indicating selection and the remaining slips indicating not being selected. Groups include between 25 and 30 participants each. All participants in the groups were included in the original sampling frame; the majority of study participants – as for group membership – were overwhelmingly women (97% in final sample). Program attrition occurred among roughly 30% of participants, largely attributed to out-migration from the county, related to sporadic rains and food insecurity. Program attrition did not vary significantly between curriculum and no-curriculum conditions. Attrition in this sample was 20% higher attrition than observed among program participants before the COVID-19 pandemic (16).

Any respondent who reported ever having tested positive for HIV was excluded from this present study but not from group membership. All respondents were 18 years of age or older.

### Curriculum exposed vs. unexposed

Consistent with the larger program goal of designing an intervention with the potential to foster flourishing communities, one cohort (*n* = 87) were pilot participants of a novel Pathways to Flourishing curriculum, while the other cohort (*n* = 111) participated in the program as usual.

### Data collection

Data were collected in Kimeru, the local language, by trained and paid nursing students with native fluency. Surveys were conducted at weekly program meetings, with groups receiving $1 per group participant. Prior focus group discussions indicated groups preferred the incentive to participate be given to the group rather than the individual respondent since individual subjects were randomly selected – ensuring everyone benefitted some from an arbitrary selection process. Data were collected using RedCap (34). T1 data were collected in November 2021, and T2 data in September–October 2022.

### Measures

The survey was first created in English using measures validated in diverse populations, translated into Kimeru (the local language), and back translated into English. Comparisons were made to the original English, and changes were made to ensure similar meanings between the Kimeru and English versions. *HIV Stigma and Discrimination*. HIV-related stigma and discrimination were measured using the 10-item “shame, blame and social isolation” subscale of the HIV stigma and discrimination scale (*α* = 0.72 for both treatments at T1; *α* = 0.73 for both treatments at T2). This sub-scale measures negative and stigmatizing attitudes toward people living with HIV and was originally developed and validated simultaneously in Zimbabwe and Thailand, and subsequently utilized and validated in Uganda, Kenya, South Africa, Tanzania and other countries across sub-Saharan Africa (35, 36). Each item is scored on a 4-point Likert-type response ranging from “strongly disagree” (1) to “strongly agree” (4), and three items are reverse scored.

#### Collective efficacy

Collective efficacy was measured using the “social response,” “social network and personal agency,” and “social attachment” subscales of the Collective Efficacy scale created by Delea et al. (37). The social response subscale included 6-items measuring perceived responsiveness of community members with statements such as “If there is a problem that affects the entire community, for instance, crop disease, people in this community will help each other.” The social network and personal agency subscale included 5-items measuring perceived presence of mutually-supporting activity between social network members, including statements like “If you and your relatives suddenly had to go away for a day or two, you could count on your neighbors to take care of your children.” The social attachment subscale included 3-items measuring the perception of being accepted by and a part of the broader social environment with statements like “people in this community accept me as a member of the community.” These 14-items were measured on a 7-point scale reflecting strength of agreement; the items have good internal reliability (*α* = 0.89 for no-curriculum and 0.77 for curriculum at T1; *α* = 0.82 for both treatments at T2).

#### Positive emotions

Positive emotions were measured using the positive emotions sub-scale of the modified Differential Emotions Scale (38). The positive emotion subscale originally included the emotion “awe” in English, but this concept does not translate culturally into Kimeru. Removing “awe” from the original English version left 9 items with good reliability at T1 and T2 (*α* = 0.81 for both treatments at T1; *α* = 0.82 for both treatments at T2). The positive emotion sub-scale includes the degree of amusement, gratitude, hope, inspiration, happiness, love, confidence, interest, and peace experienced within the past 24 h.

#### Ever HIV testing

Respondents were asked if they ever received a test for HIV from a trained healthcare provider at T2.

#### Control variables

Wealth, income, age, education and gender were recorded at T1 and included in all inferential analyses. Gender was recorded as binary – man or woman. *Wealth and income*. Wealth included household ownership of 12 items (KR20 = 0.67 at T1). Income was recorded as the self-reported average monthly income for the respondent’s household. *Age and education*. Age was recorded in years as a continuous variable. Years of formal education was likewise recorded as a continuous variable, reflecting the number of years of completed formal education.

### Statistical approach

#### Stratified and combined descriptions

We used stratified and combined analyses to compare model variables between T1 and T2, stratifying by curriculum exposure. Descriptive techniques explored model variables at T1 and T2 – including mean, 95% confidence interval. Comparison between T1 and T2 values was conducted using paired Signed Rank tests – with stratified and combined analyses. As non-negligible ceiling and floor effects were observed, respectively, for collective efficacy and HIV-related stigma, we described the main continuous variables before and after removing their most extreme value at T1. This test indicates whether there was significant difference between T1 and T2, among all respondents and respondents reporting any HIV-related stigma or less than highest collective efficacy at T1.

#### Temporal relations of primary measures

We utilized cross-lagged panel correlation analysis to assess the temporal relationship between collective efficacy, positive emotion and HIV-related stigma. Cross-lagged panel correlation analysis, an application of Structural Equation Modeling, controls for correlations between variables at each panel and for temporal correlations within each variable. Remaining significant correlations across variables at different time periods provide empirical, though not definitive, support for causal relations between variables (39). Model building for the cross-lagged panel correlation analysis followed a process beginning with a fully connected model with all control variables included. Correlations between T1 and T2 variables were removed if the corresponding value of p was less than 0.25.

#### Predictive validity of HIV stigma measure

To assess the predictive validity of the HIV-related stigma and discrimination scale, we conducted random effects logistic regression of ever HIV testing (T2) on previously reported HIV-related stigma (T1), taking into consideration all control variables. A random effect was calculated for each village. To support interpretation, the HIV-related stigma scale was standardized in logistic regression. We provide a graph showing level of T1 HIV-related stigma by T2 HIV ever-testing status, with Kruskal Wallis test of independence to compare the levels of T1 HIV-related stigma by T2 testing status.

All statistical analyses were conducted in STATA v.16 (40).

### Ethical considerations

All data were collected following written informed consent from respondents at T1 and T2. This study was provided ethical review and approval by (masked for review; IRB# 19–0241) and (masked; IRB# KeMU/SERC/21/2020).

## Results

Table 2 shows respondent characteristics and responses to variables at T1 and T2, stratified by participation in the curriculum.

**Table 2:** Stratified and combined descriptive and summative analysis of model variables.

			T1				T2					
Cohort		N	Mean (%)	SD	95% CI		Mean (%)	SD	95% CI		Sign-rank Z	*p* value
Curriculum-exposed	Collective efficacy	87	6.1	0.7	5.9	6.2	6.17	0.66	6	6.3		ns
	CE omitting highest scores (T1)	74	5.91	0.64	5.77	6.07	6.15	0.6	6	6.3	−2.5	0.01
Curriculum-unexposed	Collective efficacy	111	6	0.9	5.8	6.1	6.04	0.71	5.9	6.17		ns
	CE omitting highest scores (T1)	96	5.8	0.9	5.61	5.97	6.04	0.7	5.9	6.18	−2.4	0.02
Combined	Collective efficacy	198	6	0.84	5.89	6.13	6.1	0.7	6	6.2		ns
	CE omitting highest scores (T1)	170	5.85	0.84	5.7	5.97	6.08	0.68	6	6.19	−3.46	<0.001
Curriculum-exposed	Positive emotion	87	3	0.6	2.8	3.1	2.94	0.66	2.8	3.08		ns
	Omitting highest scores (T1)	83	2.92	0.58	2.8	3.1	2.93	0.66	2.74	3.01		ns
Curriculum-unexposed	Positive emotion	111	2.8	0.6	2.7	2.9	2.77	0.69	2.64	2.9		ns
	Omitting highest scores (T1)	111	2.8	0.6	2.7	2.9	2.77	0.69	2.65	2.88		ns
Combined	Positive emotion	198	2.89	0.64	2.8	2.98	2.8	0.7	2.7	2.9		ns
	Omitting highest scores (T1)	194	2.86	0.62	0.67	3.89	2.84	0.68	2.7	2.9		ns
Curriculum-exposed	HIV stigma & discrimination (HSD)	86	1.25	0.4	1.16	1.33	1.26	0.4	1.17	1.34		ns
	HSD Omitting lowest scores (T1)	30	1.7	0.46	1.58	1.83	1.31	0.38	1.17	1.46	4.79	<0.001
Curriculum-unexposed	HIV stigma & discrimination (HSD)	110	1.4	0.5	1.3	1.5	1.32	0.54	1.23	1.43	1.85	0.06
	HSD Omitting lowest scores (T1)	64	1.7	0.34	1.58	1.83	1.33	0.54	1.21	1.45	3.29	<0.001
Combined	HIV stigma & discrimination (HSD)	196	1.32	0.46	1.27	1.39	1.29	0.48	1.23	1.37		ns
	HSD Omitting lowest scores (T1)	92	1.7	0.43	1.61	1.79	1.32	0.49	1.22	1.41	5.84	<0.001
Combined	HIV testing, ever	198					83%	0.4	70%	90%		
Combined	Age	198					46.7	15.5	44.5	48.9		
Combined	Wealth	198					5.3	2.4	5	5.6		
Combined	Monthly income (dollars)	198					38.2	44.5	32	44.5		
Combined	Years of formal education	198					5.1	7.34	4.1	6.1		
Combined	Female gender	198					96%	0.2	93%	99%		

Descriptive analyses of T1 (Fall 2021) and T2 (Fall 2022) data. Collective efficacy (range 1–7), positive emotion (range 1–4), HIV stigma and discrimination (range 1–4) are stratified by curriculum exposed vs unexposed cohort. Wealth ranges 0–12 items on index.

The mean (SD) age of participants was 46.7 (15.5) years. The mean (SD) monthly income was $38 (44.5). The mean (SD) years of school was 5.1 (7.3). Ninety-six percent (96%) of respondents were women. The mean (SD) number of items on the wealth index was 5.3 (2.4).

**Figure 2:**
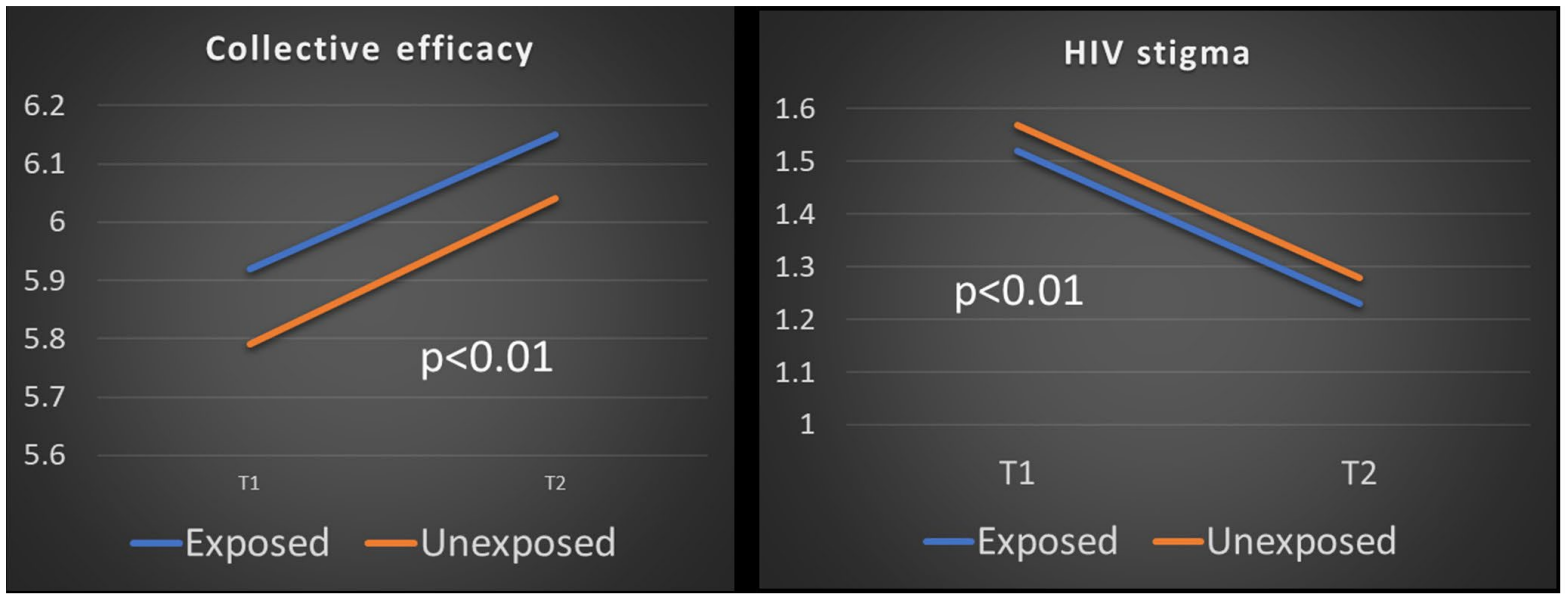
Changes in collective efficacy and HIV stigma among respondents, censoring ceiling and floor effects.

Fourteen percent of respondents reported highest level of collective efficacy at T1, with slightly higher percentage of respondents reporting highest level of T1 collective efficacy among the curriculum-exposed cohort. Nearly 45% of the curriculum-unexposed cohort, and over 65% of the curriculum-exposed cohort reported no HIV stigmatizing attitudes at T1. These ceiling and floor effects can mask potential changes among respondents with less collective efficacy or any HIV stigmatizing attitudes, respectively. After censoring ceiling effects for collective efficacy, respondents reported significantly higher collective efficacy at T2 compared to T1 in both cohorts, and combined. After censoring floor effects, respondents reported significantly less HIV stigma at T2 compared to T1 in both cohorts, and combined (*p* < 0.001) (Figure 2).

Positive emotion did not significantly vary between T1 and T2, and fewer respondents reported the highest level of positive emotion at T1 compared to collective efficacy (i.e., ceiling effects were less severe for positive emotion than collective efficacy).

At T2, 83% of respondents reported ever being tested for HIV. The mean age was 46.7 years. The mean score on the wealth index was 5.3. The mean monthly household income was $38.2. The mean years of formal education was 5.1, and 96% of respondents were women.

**Figure 3:**
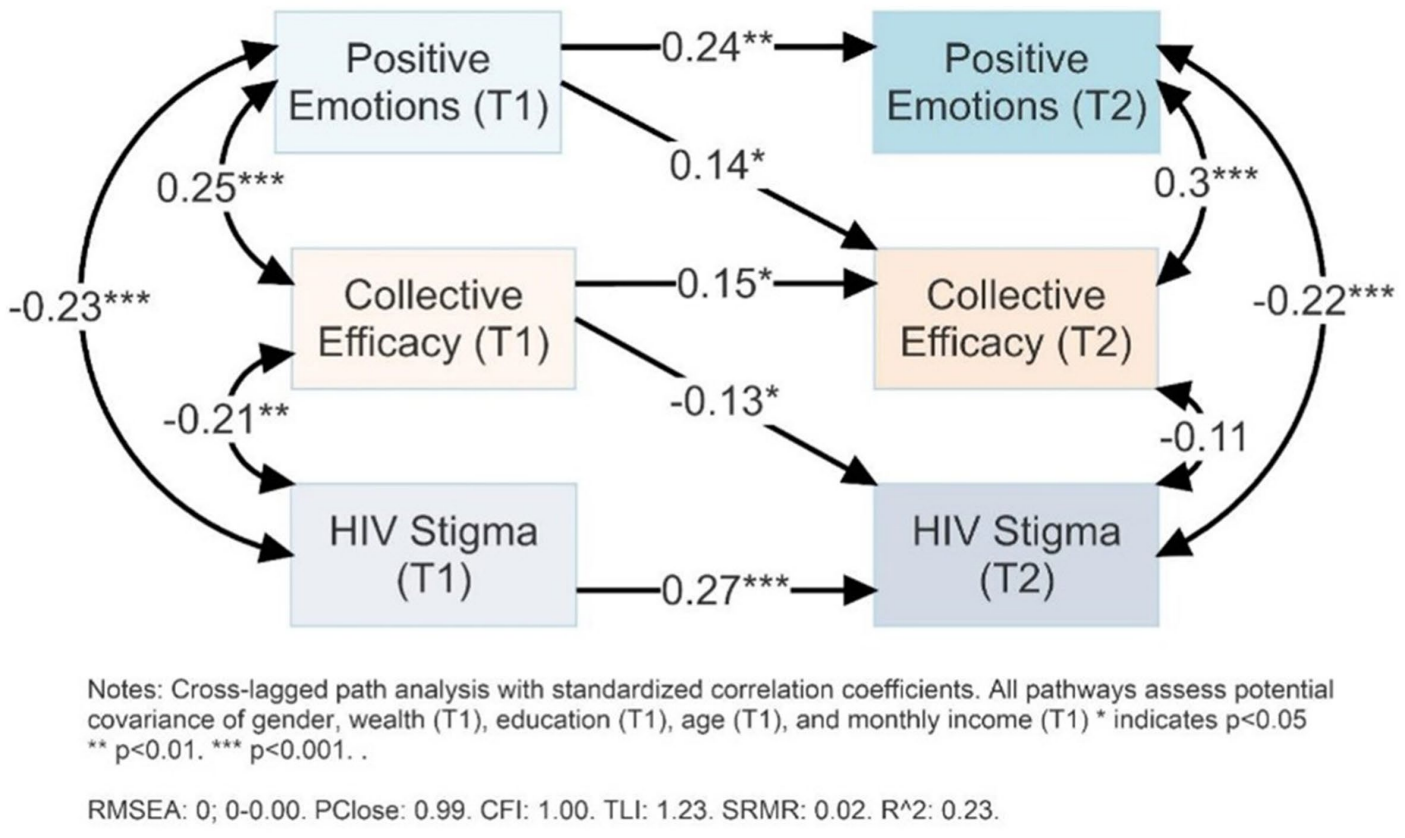
Cross-lagged panel analysis of positive emotions, collective efficacy and HIV stigma.

Figure 3 shows the cross-lagged panel analysis of positive emotion, collective efficacy, and HIV stigma and T1 and T2. The cross-lagged panel correlation analysis shows that positive emotions at T1 were significantly correlated with higher collective efficacy at T2 (*r* = 0.14, *p* < 0.05), controlling for covariates, correlations between T2 variables, and collective efficacy at T1. Collective efficacy at T1 was significantly correlated with lower HIV-related stigma at T2 (*r* = −0.13, *p* < 0.05), controlling for covariates, correlations between T2 variables, and HIV-related stigma at T1.

Table 3 shows the random effects logistic regression of ever being tested for HIV (T2) and HIV stigma (T1). As shown, the odds of reporting ever being tested at T2 decreased 28% (OR: 0.72; 95% CI: 0.54–0.96; *p* < 0.05) for each standard deviation increase in HIV stigma at T1, controlling for education, gender, age, wealth and income.

**Table 3:** Random effects logistic regression of ever HIV testing (T2) on HIV stigma (T1).

	OR	95% CI	
HIV stigma (T1, std)	0.72*	0.54	0.96
Education	1.04	0.92	1.17
Age (yrs)	0.98	0.95	1.01
Female gender	0.95	0.48	1.91
Wealth	0.98	0.78	1.22

Random effects logistic regression of ever receiving an HIV test from a trained health provider on HIV Stigma (T1), controlling for household wealth, education, age and gender. *indicates *p* < 0.05.

## Discussion

There is need for theory-based, evidence-supported, defined interventions with measurable and predicted mechanisms of action to reduce the HIV prevention policy-implementation gap. Particularly, there remain significant gaps in interventional mechanisms to (1) foster community leadership for HIV prevention, (2) reduce HIV stigma at the community-level, (3) align health systems with community-led efforts, and (4) demonstrate HIV prevention can be attained through integrating activities within communities and clinical resources. This study investigates the first two of these targets, and lays groundwork for further investigation of how the third and fourth target may be met.

Most substantially, our data show HIV stigma decreases with time in the program – significantly among participants who reported any HIV stigma at T1. This finding was true for both curriculum-exposed and -unexposed cohorts, though more pronounced for the curriculum-exposed cohort. Within this sample, this is shown to be a potentially relevant finding to HIV prevention as ever testing for HIV (T2) was predicted by significantly lower HIV stigma (T1).

These data show that community leadership, measured by collective efficacy, can increase with time in a community-located intervention. Further, later collective efficacy (T2) subsequently increases with increased positive emotion (T1) as hypothesized. During the period in which we were analyzing data for this study, participants in 19 villages – including the 11 included in this study – initiated HIV prevention resource committees. These HIV prevention resource committees aim to promote awareness of prevention approaches, including Treatment-as-Prevention (41), PrEP, HIV testing, and the community harms of HIV stigma. This development reflects part of the challenge of translating effective, community-based programming to policy and scalable implementation – the complex, ever-changing dynamics of community-led interventions. The stated intent of these HIV prevention resource committees is to promote linkage with HIV testing resources, and support subsequent linkage to care or prevention behaviors as necessary. Further documentation is required, and is beyond the scope of this study.

Further, and with greater direct relevance to HIV prevention, higher collective efficacy (T1) predicted lower HIV stigma (T2), controlling for within-time and within-variable correlations. This finding indicates the potential relevance of gender empowerment to HIV prevention, through potential impact on attitudes toward people living with HIV. The analytical design, using cross-lagged panel correlation analyses, provides the strongest possible indicator that nurturing positive emotions and collective efficacy can reduce community-level HIV stigma, until randomized control trial data demonstrate robust effectiveness and these findings are confirmed across contexts by meta-analyses.

We were surprised that neither the curriculum-exposed and curriculum-unexposed cohorts showed improved positive emotion over time, though there are at least three possible explanations. First, economic and environmental conditions were increasingly challenging during the period of this study within Kenya and East Africa. After years of relatively stable inflation rates (ranging from 4 to 5% annualized by month), inflation rates began accelerating to 6% and more in October 2021 – when T1 data were collected (42). Among other challenges, the Russian invasion of Ukraine has challenged food systems in East Africa – including Kenya (43, 44). These dynamics provide global context for reports of out-migration from study communities due to economic and food instability. It is possible that the intervention sustained positive affect, compared to a theoretical counter-factual where positive affect would otherwise decline. Sustainment of system resources, including positive affect, throughout challenges is one understanding of resilience. It is possible the intervention beneficially promotes resilience, but an appropriate comparison group is required to make this assertion. Second, it is possible that cultural-linguistic issues reduce measurement validity of the modified Differential Emotions Scale. At least one item (“awe”) did not have a separate word in the Kimeru language beyond the words translated for “interest” and “inspired,” reducing the number of items from 10 to 9. However, internal consistency was high in all survey waves and cohorts, the measure was stable within individuals after a year, and predicted subsequently strengthened collective efficacy as hypothesized. Thus, while it is unlikely that measurement issues alone produced the observed non-improvement, further investigation into emotional states and measures is necessary within non-English speaking populations. Finally, it is possible that the intervention did not have any impact on positive emotion – neither through sustaining positive affect in a challenging time nor identifiable through other potential measures. While this interpretation is not consistent with observed improvements to collective efficacy within this study, or improved depression within parallel studies (22), the question of for whom the intervention works and through what interventional, economic, social, and psychological mechanisms requires further research.

We were surprised to see collective efficacy and HIV stigma reduced in both curriculum-exposed and -unexposed cohorts. Future research should ensure an adequately large sample, and stratify randomization to ensure the curriculum-exposed villages have even matches with curriculum-unexposed and waitlist control villages. It is possible the curriculum does not add measurable value to participants, though this conflicts with on-going programmatic qualitative assessments. The study included a feasibility study of the curriculum, and was not designed, or powered, to support a direct comparison between the two cohorts.

The broader scientific context of this intervention emerges from research on cultural evolution studies and multilevel selection theory. These broader scientific strains provide emerging consensus that the capacity for groups to cooperate, coordinate activity, and nurture member families and individuals is fundamental to human flourishing (45). At individual-levels, the capacity to vary, select and retain affective, cognitive, motivational, attentional and overt behavioral states drives individual-level health adaptation to changing environments (24). Fostering community-, group-, and individual-level conditions that support engagement and leadership in HIV prevention will be an essential part of implementing the global HIV policy vision. We assert this flourishing community intervention includes components that can improve these multi-level conditions, and warrants further experimental investigation to clarify its effectiveness, simplicity, adaptability, scalability, and systems-level adoption.

With respect to reducing the HIV prevention policy-implementation gap, and achieving the goals set out in the UNGA and Kenya NASCOP policy documents, this intervention demonstrates potential to (1) increase community leadership, and (2) decrease HIV stigma at a community-scale. We have sought to define mechanisms of action, and theoretical support, to promote future development and modification of this intervention. Simplifying the complexity involved in bottom-up, experimental implementations of policy is necessary for system-level adoption. We have attempted to demonstrate interventional actions and mechanisms of change that can foster community leadership, gender empowerment, reduce HIV stigma and prepare for deeper integration with clinical infrastructure.

### Limitations

Internal and external validity of this study are threatened by two key sources. First, all data are self-reported data. While psychometric assessments demonstrate internal and predictive reliability, other measurement sources (e.g., observed uptake of HIV testing; confirmed fewer experiences of HIV stigma experiences by people living with HIV) are required to confirm findings. Self-reported data are subject to recall- and social desirability-biases. Second, all data are from intervention participants and variations between T1 and T2 measures may reflect unmeasured and unaccounted for environmental or routine temporal changes. Intervention and implementation research is required to further consideration of this promising approach to the level of systems-level adoption. Also, further experimental implementation is required to reduce the HIV prevention policy-implementation gap, and the flourishing community model presents an enticing direction.

Another limitation of this study is that the curriculum was delivered, by random allocation, to communities reporting higher positive affect, collective efficacy and lower HIV stigma compared to the curriculum-unexposed communities. Future research should stratified randomization to curriculum or no curriculum conditions.

## Conclusion

Synthesizing recent trends within behavioral and evolutionary science within a community-based intervention, we find interventional efficacy to improve collective efficacy and reduce community-level HIV-related stigma. Reducing community-level HIV stigma is essential to turning the corner on the HIV pandemic, including the need for increased HIV testing. While further research is required, articulating the mechanisms by which complex interventions achieve various outcomes remains essential for external validation and systems-level adoption. Further research can build on this study by demonstrating effectiveness with proper control comparisons, and observing improvements in behavioral adoption of HIV testing, prevention and treatment.

## Data availability statement

The raw data supporting the conclusions of this article will be made available by the authors, without undue reservation.

## Ethics statement

The studies involving humans were approved by Kenya Methodist University and University of Texas Medical Branch. The studies were conducted in accordance with the local legislation and institutional requirements. The participants provided their written informed consent to participate in this study.

## Author contributions

MG conceived of and led the study, writing, and analysis. JT provided conceptual support and editorial contributions. PK provided subject matter expertise and policy analysis. SS provided editorial revisions. LR-G provided conceptual support and editorial support. SG led the intervention and management within Kenya. FM provided expertise in data collection and interpretation. MB provided thematic expertise and editorial revision. PP provided thematic support, editorial support, and funding support.

## References

[1] United Nations General Assembly. Political Declaration on HIV and AIDS: ending inequalities and getting on track to end AIDS by 2030. (2021). Available at: https://www.unaids.org/en/resources/documents/2021/2021_political-declaration-on-hiv-and-aids.

[2] National AIDS and STI Control Program. Kenya population-based HIV impact assessment (KENPHIA) 2018: Final report. Nairobi: NASCOP (2022).

[3] Camlin CS Charlebois ED Getahun M Akatukwasa C Atwine F Itiakorit H. Pathways for reduction of HIV-related stigma: a model derived from longitudinal qualitative research in Kenya and Uganda. J Int AIDS Soc. (2020) 23:e25647. doi: 10.1002/jia2.25647, PMID: 33283986 PMC7720278

[4] Nyblade L Mingkwan P Stockton MA. Stigma reduction: an essential ingredient to ending AIDS by 2030. Lancet HIV. (2021) 8:e106–13. doi: 10.1016/S2352-3018(20)30309-X, PMID: 33539757

[5] Sullivan MC Rosen AO Allen A Benbella D Camacho G Cortopassi AC. Falling short of the first 90: HIV stigma and HIV testing research in the 90–90–90 era. AIDS Behav. (2020) 24:357–62. doi: 10.1007/s10461-019-02771-7, PMID: 31907675

[6] Kalichman SC Shkembi B Wanyenze RK Naigino R Bateganya MH Menzies NA. Perceived HIV stigma and HIV testing among men and women in rural Uganda: a population-based study. Lancet HIV. (2020) 7:e817–24. doi: 10.1016/S2352-3018(20)30198-3 32910903 PMC9706443

[7] Moran A Mashele N Mvududu R Gorbach P Bekker LG Coates TJ. Maternal PrEP use in HIV-uninfected pregnant women in South Africa: role of stigma in PrEP initiation, retention and adherence. AIDS Behav. (2022) 26:205–217. doi: 10.1007/s10461-021-03374-x 34287756 PMC8294266

[8] Calabrese SK Mayer KH. Stigma impedes HIV prevention by stifling patient–provider communication about U= U. J Int AIDS Soc. (2020) 23:559. doi: 10.1002/jia2.25559 PMC7369401 32686324

[9] Thornhill J Orkin C. Long-acting injectable HIV therapies: the next frontier. Curr Opin Infect Dis. (2021) 34:8–15. doi: 10.1097/QCO.0000000000000701, PMID: 33337617

[10] Hudson B Hunter D Peckham S. Policy failure and the policy-implementation gap: can policy support programs help? Policy Design Prac. (2022) 2:1–4. doi: 10.1080/25741292.2018.1540378

[11] Kawonga M Blaauw D Fonn S. Aligning vertical interventions to health systems: a case study of the HIV monitoring and evaluation system in South Africa. Heal Res Policy Syst. (2012) 10:1–3. doi: 10.1186/1478-4505-10-2 PMC3293072 22280794

[12] Zakumumpa H Rujumba J Kwiringira J Kiplagat J Namulema E Muganzi A. Understanding the persistence of vertical (stand-alone) HIV clinics in the health system in Uganda: a qualitative synthesis of patient and provider perspectives. BMC Health Serv Res. (2018) 18:1–3. doi: 10.1186/s12913-018-3500-4 30185191 PMC6126041

[13] Mussa AH Pfeiffer J Gloyd SS Sherr K. Vertical funding, non-governmental organizations, and health system strengthening: perspectives of public sector health workers in Mozambique. Hum Resour Health. (2013) 11:1–9. doi: 10.1186/1478-4491-11-26, PMID: 23768178 PMC3691708

[14] Assefa Y Gilks CF. Ending the epidemic of HIV/AIDS by 2030: will there be an endgame to HIV, or an endemic HIV requiring an integrated health systems response in many countries? Int J Infect Dis. (2020) 100:273–7. doi: 10.1016/j.ijid.2020.09.011, PMID: 32920236

[15] Das P Horton R. Beyond the silos: integrating HIV and global health. Lancet. (2018) 392:260–1. doi: 10.1016/S0140-6736(18)31466-1, PMID: 30032981

[16] Parkhurst JO Hunsmann M. Breaking out of silos–the need for critical paradigm reflection in HIV prevention. Rev African Political Econ. (2015) 42:477–87. doi: 10.1080/03056244.2015.1064373

[17] Goodman M Theron L Seidel S Elliott A Raimer-Goodman L Keiser P. Flourishing communities: a new model to promote sustainable community leadership and transformation in semi-rural Kenya. J Community Appl Soc Psychol. (2023) 33:756–72. doi: 10.1002/casp.2665 37213894 PMC10195071

[18] Goodman ML Elliott AJ Gitari S Keiser P Raimer-Goodman L Seidel SE. Come together to promote health: case study and theoretical perspectives from a Kenyan community-based program. Health Promot Int. (2021) 36:1765–74. doi: 10.1093/heapro/daab018, PMID: 33604649 PMC13381515

[19] Cox J Raimer-Goodman L Gatwiri C Elliott A Goodman M. Partner cooperation, conflict, maternal mental health, and parenting Behaviors in rural Kenya: towards a two-generational understanding of gender transformation benefits. Int J Child Maltreatment: Res Policy Pract. (2023):1–2. doi: 10.1007/s42448-023-00156-x PMC10852061 38333764

[20] Goodman ML Elliott AJ Gitari S Keiser P Onwuegbuchu E Michael N. Come together to decrease depression: Women’s mental health, social capital, and participation in a Kenyan combined microfinance program. Int J Soc Psychiatry. (2021) 67:613–21. doi: 10.1177/0020764020966014, PMID: 33059496 PMC8050113

[21] Goodman ML Elliott A Melby PC Gitari S. Water insecurity, food insecurity and social capital associated with a group-led microfinance programme in semi-rural Kenya. Glob Public Health. (2022) 17:3399–411. doi: 10.1080/17441692.2022.2095656 35787237 PMC9810762

[22] Goodman ML Temple JR Elliott AJ Seidel SE Gitari S Raimer-Goodman LA. Child maltreatment, social capital, maternal mental health and duration of program involvement: assessment from a community-based empowerment program in Kenya. J Family Violence. (2022) 38:407–17. doi: 10.1007/s10896-022-00391-9 PMC10187605 37197413

[23] Goodman ML Seidel SE Springer A Elliott A Markham C Serag H. Enabling structural resilience of street-involved children and youth in Kenya: reintegration outcomes and the flourishing community model. Front Psychol. (2023) 14:593. doi: 10.3389/fpsyg.2023.1175593, PMID: 37680240 PMC10482225

[24] Hayes SC Hofmann SG Ciarrochi J. A process-based approach to psychological diagnosis and treatment: the conceptual and treatment utility of an extended evolutionary meta model. Clin Psychol Rev. (2020) 82:101908. doi: 10.1016/j.cpr.2020.101908, PMID: 32932093 PMC7680437

[25] O'Gorman R Sheldon KM Wilson DS. For the good of the group? Exploring group-level evolutionary adaptations using multilevel selection theory. Group Dyn Theory Res Pract. (2008) 12:17–26. doi: 10.1037/1089-2699.12.1.17

[26] Hayes S. C. Hofmann S. G. Ciarrochi J. Building a process-based diagnostic system: An extended evolutionary approach. Beyond the DSM: Toward a process-based alternative for diagnosis and mental health treatment. (2020):251–278.

[27] Dovidio JF Piliavin JA Schroeder DA Penner LA. The social psychology of prosocial behavior. UK: Psychology Press (2017). 10.1146/annurev.psych.56.091103.070141 15709940

[28] Wilson DS Hayes SC Biglan A Embry DD. Evolving the future: toward a science of intentional change. Behav Brain Sci. (2014) 37:395–416. doi: 10.1017/S0140525X13001593, PMID: 24826907 PMC4331065

[29] Mikulincer M Shaver PR. Enhancing the “broaden-and-build” cycle of attachment security as a means of overcoming prejudice, discrimination, and racism. Attach Hum Dev. (2022) 24:260–73. doi: 10.1080/14616734.2021.1976921, PMID: 34499022

[30] Mikulincer M Shaver PR. Enhancing the “broaden and build” cycle of attachment security in adulthood: from the laboratory to relational contexts and societal systems. Int J Environ Res Public Health. (2020) 17:2054. doi: 10.3390/ijerph17062054, PMID: 32244872 PMC7143531

[31] Emery M Flora C. Spiraling-up: mapping community transformation with community capitals framework. Community Dev. (2006) 37:19–35. doi: 10.1080/15575330609490152

[32] Fredrickson BL Joiner T. Reflections on positive emotions and upward spirals. Perspec Psychol Sci. (2018) 13:194–9. doi: 10.1177/1745691617692106, PMID: 29592643 PMC5877808

[33] Lykes MB. Participatory and action research as a transformative praxis: responding to humanitarian crises from the margins. Am Psychol. (2013) 68:774–83. doi: 10.1037/a0034360, PMID: 24320677

[34] Harris PA Taylor R Minor BL Elliott V Fernandez M O'Neal L. The REDCap consortium: building an international community of software platform partners. J Biomed Inform. (2019) 95:103208. doi: 10.1016/j.jbi.2019.103208 31078660 PMC7254481

[35] Genberg BL Kawichai S Chingono A Sendah M Chariyalertsak S Konda KA. (2008). Assessing HIV/AIDS stigma and discrimination in developing countries. AIDS Behav. 12:772–780. 18080830 10.1007/s10461-007-9340-6 PMC2745205

[36] Turan JM Bukusi EA Onono M Holzemer WL Miller S Cohen CR (2011). HIV/AIDS stigma and refusal of HIV testing among pregnant women in rural Kenya: results from the MAMAS Study. AIDS Behav. 15, 1111–1120. 20827573 10.1007/s10461-010-9798-5 PMC3127002

[37] Delea MG Sclar GD Woreta M Haardörfer R Nagel CL Caruso BA. (2018). Collective efficacy: development and validation of a measurement scale for use in public health and development programmes. Int J Environ Res Public Health. 15:2139. 30274212 10.3390/ijerph15102139 PMC6211028

[38] Fredrickson BL. (2013). Positive emotions broaden and build. In Advances in experimental social psychology (Vol. 47) Academic Press. pp. 1–53.

[39] Sussman R Gifford R. (2019). Causality in the theory of planned behavior. Pers Soc Psychol Bull. 45, 920–933. 30264655 10.1177/0146167218801363

[40] StataCorp. (2019). Stata Statistical Software: Release 16. College Station, TX: StataCorp LLC.

[41] Boyer S Iwuji C Gosset A Protopopescu C Okesola N Plazy M. Factors associated with antiretroviral treatment initiation amongst HIV-positive individuals linked to care within a universal test and treat programme: early findings of the ANRS 12249 TasP trial in rural South Africa. AIDS Care. (2016) 28:39–51. doi: 10.1080/09540121.2016.1164808, PMID: 27421051 PMC5096681

[42] Central Bank of Kenya. Inflation rates. (2023). Available at: https://www.centralbank.go.ke/inflation-rates/, accessed, 2023.

[43] McGuirk E Burke M. War in Ukraine, world food prices, and conflict in Africa. Africa: Global Economic Consequences of the War in Ukraine Sanctions, supply chains and sustainability (2022).

[44] Laganda G. Responding to loss and damage in food systems. Nature Food. (2023) 4:133–4. doi: 10.1038/s43016-023-00702-3, PMID: 37117854

[45] Biglan A Flay BR Embry DD Sandler IN. The critical role of nurturing environments for promoting human well-being. Am Psychol. (2012) 67:257–71. doi: 10.1037/a0026796, PMID: 22583340 PMC3621015

